# Adjuvant therapy for cholangiocarcinoma after surgery and prognosis factors for cholangiocarcinoma: A single-center retrospective cohort study

**DOI:** 10.3389/fonc.2023.1116338

**Published:** 2023-03-17

**Authors:** Zhongquan Sun, Xin Han, Wanlu You, Jinlong Tang, Juehua Xu, Binglin Ye, Tengfei Li, Yixin Zhang, Kai Chen, Yuan Ding, Weilin Wang

**Affiliations:** ^1^ Department of Hepatobiliary and Pancreatic Surgery, The Second Affiliated Hospital, Zhejiang University School of Medicine, Hangzhou, Zhejiang, China; ^2^ Key Laboratory of Precision Diagnosis and Treatment for Hepatobiliary and Pancreatic Tumor of Zhejiang Province, Hangzhou, Zhejiang, China; ^3^ Research Center of Diagnosis and Treatment Technology for Hepatocellular Carcinoma of Zhejiang Province, Hangzhou, Zhejiang, China; ^4^ National Innovation Center for Fundamental Research on Cancer Medicine, Hangzhou, Zhejiang, China; ^5^ Cancer Center, Zhejiang University, Hangzhou, Zhejiang, China; ^6^ Zhejiang University (ZJU)-Pujian Research & Development Center of Medical Artificial Intelligence for Hepatobiliary and Pancreatic Disease, Hangzhou, Zhejiang, China; ^7^ Department of Pathology, The Second Affiliated Hospital, Zhejiang University School of Medicine, Hangzhou, Zhejiang, China

**Keywords:** adjuvant therapy, cholangiocarcinoma, surgery, early stage, prognosis factors

## Abstract

**Background:**

Cholangiocarcinoma (CCA) is a highly heterogeneous malignant tumor, and more than 60% of patients have recurrence and metastasis after surgery. The efficacy of postoperative adjuvant therapy for CCA remains unclear. This study aimed to explore whether adjuvant therapy benefits patients with CCA and examine the independent prognostic factors for overall survival (OS) and progression-free survival (PFS).

**Methods:**

Patients with CCA undergoing surgery were retrospectively enrolled in this study from June 2016 to June 2022. The chi-square test or Fisher exact test was used to analyze the correlation between clinicopathologic characteristics. Survival curves were plotted using the Kaplan-Meier method, and the Cox regression model was used for univariate and multivariate analysis to search for independent prognostic factors.

**Results:**

Of the 215 eligible patients, 119 patients received adjuvant therapy, and the other 96 patients did not. The median follow-up was 37.5 months. The median OS of CCA patients with and without adjuvant therapy was 45 and 18 months (*P* < 0.001), respectively. The median PFS of CCA patients with and without adjuvant therapy was 34 and 8 months (*P* < 0.001), respectively. The Cox univariate and multivariate regression analysis showed that preoperative aspartate transaminase and carbohydrate antigen 19-9, microvascular invasion, lymph node metastasis, differentiation degree, and adjuvant therapy were independent prognostic factors for OS (all *P* values < 0.05). Preoperative carbohydrate antigen 125, microvascular invasion, lymph node metastasis, differentiation degree, and adjuvant therapy were independent prognostic factors for PFS (all *P* values < 0.05). The stratified analysis by TMN stage detected significant differences in the early stages (median OS [mOS]: *P *= 0.0128; median PFS [mPFS]: *P *= 0.0209) and advanced stages (mOS and mPFS: both *P* values < 0.001). Adjuvant therapy was also identified as a significantly favorable prognostic factor for OS and PFS in the early stages and advanced stages.

**Conclusion:**

Postoperative adjuvant therapy can improve the prognosis of patients with CCA, even in the early stages and advanced stages. All data suggest that adjuvant therapy should be incorporated into the treatment of CCA in all cases, where appropriate.

## Introduction

Cholangiocarcinoma (CCA) is a highly lethal epithelial cell malignancy emerging in the hepatobiliary system, which can be divided into 3 subtypes based on its anatomical location: intrahepatic CCA (iCCA), perihilar CCA (pCCA), and distal CCA (dCCA) (1–3). Together, CCA represents approximately 15% of all primary liver tumors and 3% of all instances of gastrointestinal neoplasia (4, 5). Despite being rare, the incidence of CCA worldwide has been increasing in the past few decades (4). CCA has no specific symptoms in the early stages and is highly malignant; therefore, more than 70% of CCA are diagnosed in the advanced stages, and CCA has become a global health burden (4–6).

Surgery is the only potentially curative treatment for patients with CCA (7, 8). When the disease is resectable, the patients’ median overall survival (mOS) can surpass 40 months, and the 5-year OS rate is 25–40% (9–11). However, more than 60% of patients with CCA relapse after surgery, which has led several studies to explore the use of adjuvant therapy for CCA (4, 11). Recently, 3 phase III randomized clinical studies were reported to inform practice. The PRODIGE 12 study randomly assigned patients with CCA or gallbladder cancer to surgery alone or surgery followed by 6 months of gemcitabine and oxaliplatin (12). Furthermore, in the BCAT study, patients with pCCA or dCCA were randomly assigned to surgery alone or surgery followed by 6 months of gemcitabine (13). Disappointingly, both these studies did not meet their primary endpoint. In contrast, the BILCAP study revealed a significant benefit in OS from adjuvant capecitabine in prespecified sensitivity and per-protocol analysis when compared with observation alone (hazard ratio [HR] 0.71; *P* = 0.010) (14). In addition, the role of postoperative adjuvant therapy for CCA remains uncertain, and there are conflicting results reported from nonrandomized and randomized studies (15). Therefore, there is a need for further study to explore the efficacy of adjuvant therapy after surgery in people with CCA.

In this study, we retrospectively enrolled patients with CCA undergoing surgery in the Second Affiliated Hospital, Zhejiang University School of Medicine from June 2016 to June 2022. First, we investigated the efficacy of adjuvant therapy for CCA. Second, independent prognostic factors of CCA after surgery were examined. As the novel aspect of our study, we further divided all patients into early-stage or advanced-stage groups to evaluate the beneficial effect of postoperative adjuvant therapy for patients in these 2 subgroups.

## Methods

### Patients selection

A retrospective analysis was performed on all patients undergoing curative resection for histologically confirmed CCA in the Second Affiliated Hospital, Zhejiang University School of Medicine between June 2016 and June 2022. Patients were included if they (1) underwent radical resection and had a pathological diagnosis of CCA; and (2) did not receive local radiotherapy or systemic chemotherapy, targeted therapy, immunotherapy, cancer vaccine, or other treatments for any purpose before surgery. Patients were excluded if they (1) had incomplete clinical data; (2) had a history of other malignant tumors, stroke, acute coronary syndrome, autoimmune diseases, systemic infectious diseases, multiple organ dysfunction, etc; (3) experienced perioperative death; or (4) experienced major trauma or other surgery within 3 months. The judging criteria included pathological diagnostic criteria and staging criteria. (1) The pathological diagnostic criteria were those found in the *WHO Classification Tumors of the Digestive System, 4^th^ Edition* issued by the International Agency for Research on Cancer (IARC) in 2010. (2) The staging criteria were the TNM staging for CCA according to the eighth edition of the American Joint Committee on Cancer (AJCC)/International Union Against Cancer (UICC).

The study was conducted in accordance with the ethical guidelines of the 1975 Declaration of Helsinki. All patients had signed written informed consent. Ethical approval was obtained from the Ethics Committee of the Second Affiliated Hospital, Zhejiang University School of Medicine.

### Data collection

All clinical data were retrospectively obtained from medical records and included age, gender, body mass index (BMI), albumin (ALB), alanine transaminase (ALT), aspartate transaminase (AST), carbohydrate antigen 19-9 (CA19-9), carbohydrate antigen 125 (CA125), pathological type, resection margin, microvascular invasion, lymph node metastasis, differentiation degree, TNM stage, and adjuvant chemotherapy regimen. The primary endpoints were overall survival (OS) and progression-free survival (PFS). OS was defined as the time from tumor diagnosis to follow-up or death, and PFS was defined as the time from tumor diagnosis to disease recurrence or death.

All data collection and analysis were reviewed by 2 and more senior clinicians.

### Follow-up

Follow-up was performed in the outpatient clinic or by telephone. Blood routine, blood biochemistry, hepatorenal function, tumor biomarkers, and computed tomography (CT) or magnetic resonance imaging (MRI) examinations were detected every 3 months within 1 year after surgery and then every 6 months for more than 1 year after surgery. All patients were followed up until September 2022.

### Statistical analysis

All data were analyzed using SPSS 19.0 (IBM Corp., Armonk, NY, USA). Continuous variables are described as the mean ± standard deviation, while categorical variables are expressed as a frequency. Continuous variables were assessed with the Student *t* test, while the chi-square test and Fisher exact test were used to analyze categorical variables. The Kaplan-Meier method was applied to the calculate survival curve, and the Cox regression model was conducted for univariate and multivariate analysis. GraphPad Prism version 8.0 (GraphPad Software, San Diego, CA, USA) was used to draw survival analysis curves. A *P* value < 0.05 was considered statistically significant.

## Results

**Figure 1:**
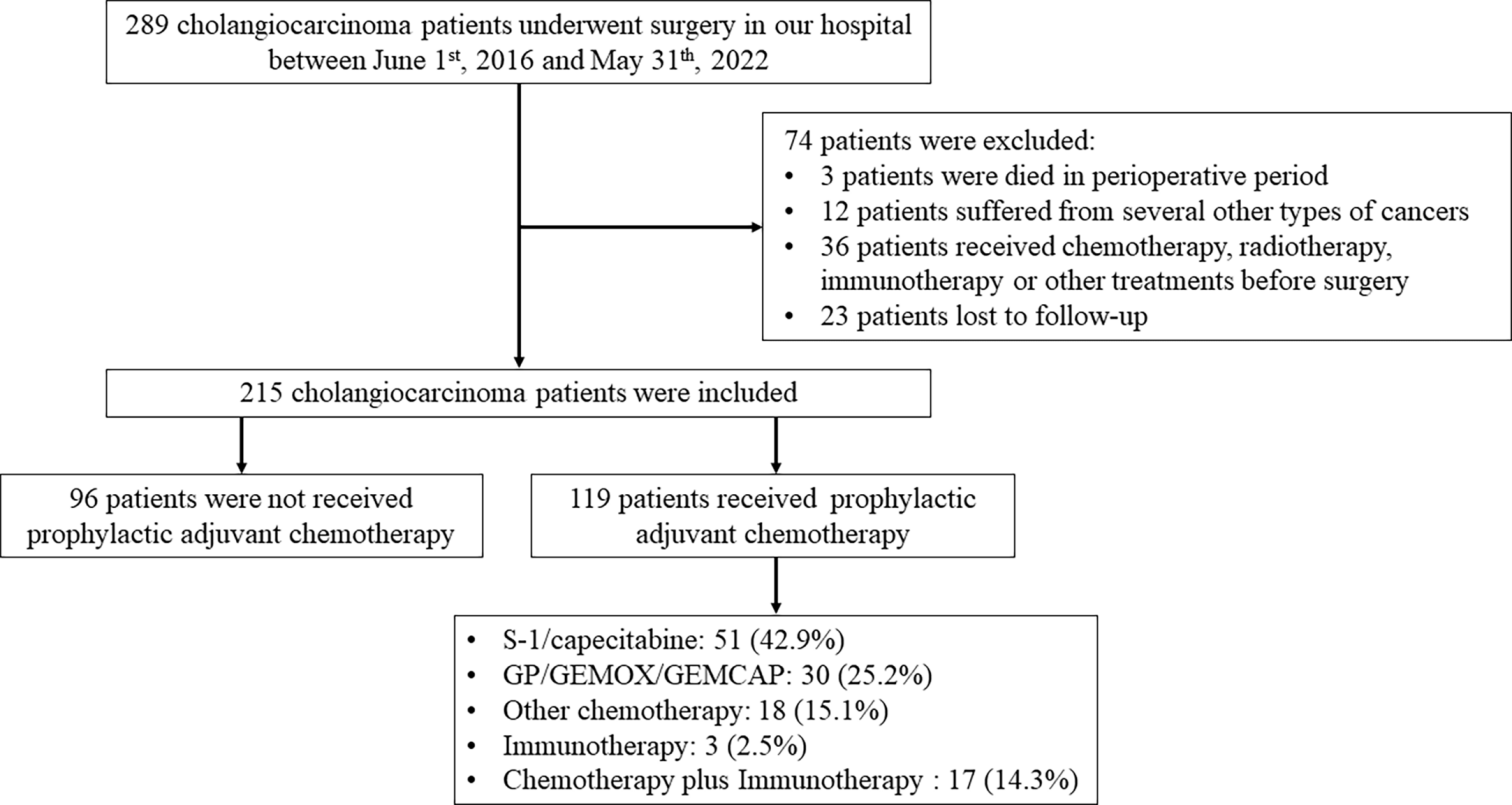
Workflow of our study.

As depicted in **Figure 1**, 215 patients undergoing surgery for pathologically confirmed CCA between June 2016 and June 2022 were considered for inclusion. A total of 119 cases received postoperative adjuvant therapy, while 96 cases did not. A total of 51 (42.9%) patients received oral S-1 or capecitabine, which was the main adjuvant therapy regimen in our study. According to **Table 1**, the average age of all patients was 62.34 ± 10.05 years and most patients are male. The number of iCCA, pCCA and dCCA was 147, 63 and 5; while patients on stage I, II, III, IV were 64, 56, 68, 27, respectively.

**Table 1:** Basic clinicopathologic characteristics.

Basic characteristics	n
Age (years)	62.34±10.05
Sex (male / female)	115/100
BMI (kg/m^2^)	22.79±3.10
Type (iCCA / pCCA / dCCA)	147/63/5
AJCC 8th edition Stage (I / II / III / IV)	64/56/68/27

### Clinicopathologic characteristics and survival analysis in all patients

As shown in **Table 2**, there were no significant differences between the adjuvant therapy and non-adjuvant therapy groups in age (*P *= 0.073), gender (*P *= 0.711), BMI (*P *= 0.764), albumin (*P *= 0.329), ALT (*P *= 0.607), AST (*P *= 0.642), CA19-9 (*P *= 0.072), CA125 (*P *= 0.349), pathological type (*P *= 0.527), margin status (*P *= 0.839), microvascular invasion (*P *= 0.704), lymph node metastasis (*P *= 0.452), differentiation degree (*P *= 0.855), or TNM stage (*P *= 0.206).

**Table 2:** Clinicopathologic characteristics in all patients.

Characteristics	Adjuvant therapy (n=119)	Non- adjuvant therapy (n=96)	P
Age (years)			0.073
≤60	54	32	
>60	65	64	
Sex			0.711
Male	65	50	
Female	54	46	
BMI (kg/m^2^)			0.764
<24.0	77	64	
≥24.0	42	32	
Albumin (g/dL)			0.329
<3.5	22	13	
≥3.5	97	83	
ALT (U/L)			0.607
<40	64	55	
≥40	55	41	
AST (U/L)			0.642
<40	67	51	
≥40	52	45	
CA19-9 (U/mL)			0.072
<40	63	39	
≥40	56	57	
CA125 (U/mL)			0.349
<35	91	68	
≥35	28	28	
Pathological Type			0.527
Intrahepatic	80	67	
Perihilar	35	28	
Distal	4	1	
Resection Margin			0.839
R0	104	83	
R1	15	13	
Microvascular Invasion			0.704
Yes	49	42	
No	70	54	
Lymph Node Metastasis			0.452
Yes	34	32	
No	85	64	
Differentiation Degree			0.855
Poor	54	40	
Moderate	54	47	
Well	11	9	
AJCC 8th edition Stage			0.206
I/II	71	49	
III/IV	48	47	

BMI, Body Mass Index; ALT, Alanine Transaminase; AST, Aspartate Transaminase; CA19-9, Carbohydrate Antigen 19-9; CA125, Carbohydrate Antigen 125.

**Figure 2:**
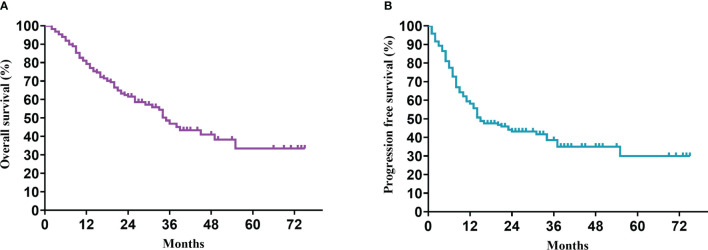
Kaplan-Meier curves of all patients with CCA after curative resection. **(A)** OS; **(B)** PFS.

**Figure 3:**
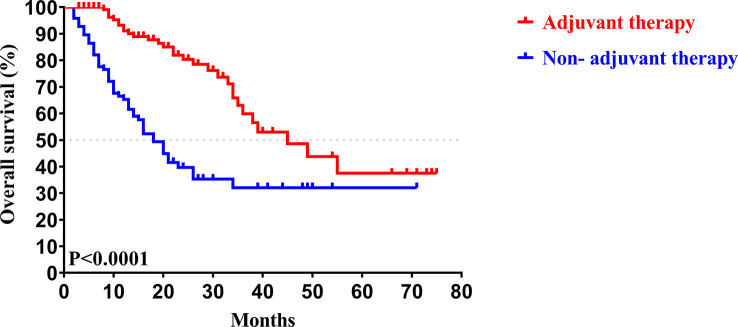
Kaplan-Meier OS curves of patients with and without adjuvant therapy after curative resection.

**Figure 4:**
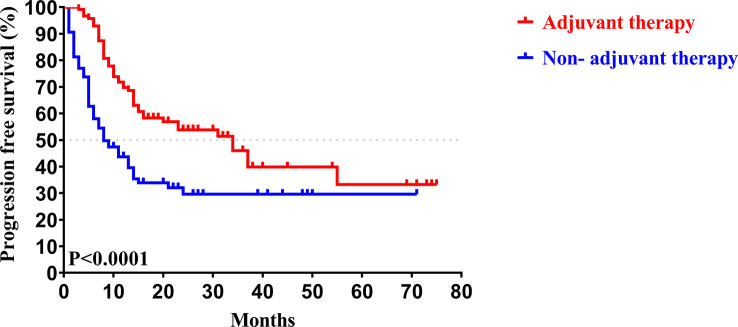
Kaplan-Meier PFS curves of patients with and without adjuvant therapy after curative resection.

In our study, the median follow-up time was 37.5 months. The 1-, 3-, and 5-year OS rates of all patients with CCA after surgery were 79.34%, 46.94%, and 33.40%, respectively, while the 1-, 3-, and 5-year PFS rates were 58.14%, 38.50%, and 30.00%, respectively (**Figure 2**). We further explored the survival difference between the adjuvant therapy and non-adjuvant therapy groups. For the patients with and without adjuvant therapy, the results showed that the median OS was 45 and 18 months (*P* < 0.001; **Figure 3**), respectively, and the median PFS was 34 and 8 months (*P* < 0.001; **Figure 4**), respectively. Therefore, postoperative adjuvant therapy may greatly benefit patients with CCA.

### Univariate and multivariate analysis of OS and PFS in all patients

We then performed univariate and multivariate analyses of OS and PFS to find the prognostic factors for all patients with CCA after surgical resection. **Table 3** shows that preoperative AST (HR = 1.703; *P *= 0.022), CA19-9 (HR = 1.646; *P *= 0.040), microvascular invasion (HR = 1.632; *P *= 0.031), lymph node metastasis (HR = 2.983; *P* < 0.001), differentiation degree (HR = 2.429; *P* < 0.001), and adjuvant therapy (HR = 4.392; *P* < 0.001) were independent prognostic factors for OS. Meanwhile, **Table 4** shows that preoperative CA125 (HR = 1.585; *P *= 0.030), microvascular invasion (HR = 1.722; *P *= 0.007), lymph node metastasis (HR=3.194; *P* < 0.001), differentiation degree (*P *= 0.008; HR = 1.704), and adjuvant therapy (HR = 2.922; *P *< 0.001) were independent prognostic factors for PFS.

**Table 3:** Univariate and multivariate analysis of overall survival in all patients.

Characteristics	Univariate analysis		Multivariate analysis	
	HR	P	HR	P
Age (years)	0.951	0.820		
>60 vs ≤60	(0.614-1.473)			
Sex	0.736	0.167		
Male vs Female	(0.476-1.136)			
BMI (kg/m^2^)	1.220	0.379		
≥40 vs <24.0	(0.783-1.899)			
Albumin (g/dL)	1.390	0.218		
<3.5 vs ≥3.5	(0.823-2.346)			
ALT (U/L)	1.380	0.143		
≥40 vs <40	(0.897-2.124)			
AST (U/L)	**1.470**	**0.081**	**1.703**	0.022*
≥40 vs <40	**(0.953-2.268)**		**(1.079-2.687)**
CA19-9 (U/mL)	**2.114**	0.001**	**1.646**	0.040*
≥40 vs <40	**(1.346-3.319)**		**(1.023-2.648)**	
CA125 (U/mL)	1.778	0.013*		
≥35 vs <35	(1.127-2.805)			
Resection Margin	1.821	0.028*		
R1 vs R0	(1.067-3.108)			
Microvascular Invasion	**1.875**	0.005**	**1.632**	0.031*
Yes vs No	(1.211-2.901)		(1.045-2.551)	
Lymph Node Metastasis	**3.248**	<0.001**	**2.983**	<0.001**
Yes vs No	(2.080-5.071)		(1.847-4.819)	
Differentiation Degree	**2.082**	0.001**	**2.429**	<0.001**
Poor vs Moderate and Well	(1.332-3.254)		(1.498-3.939)	
Adjuvant therapy	**3.103**	<0.001**	**4.392**	<0.001**
No vs Yes	(1.975-4.874)		(2.706-7.129)	

BMI, Body Mass Index; ALT, Alanine Transaminase; AST, Aspartate Transaminase; CA19-9, Carbohydrate Antigen 19-9; CA125, Carbohydrate Antigen 125. *P<0.05, **P<0.01. The bold values mean that these characteristics have statistical significance. In brief, the P value of these characteristics <0.05.

**Table 4:** Univariate and multivariate analysis of progression free survival in all patients.

Characteristics	Univariate analysis		Multivariate analysis	
	HR	P	HR	P
Age (years)	0.845	0.380		
>60 vs ≤60	(0.579-1.231)			
Sex	1.008	0.966		
Male vs Female	(0.693-1.466)			
BMI (kg/m^2^)	1.300	0.177		
≥40 vs <24.0	(0.888-1.903)			
Albumin (g/dL)	1.036	0.887		
<3.5 vs ≥3.5	(0.637-1.683)			
ALT (U/L)	1.042	0.830		
≥40 vs <40	(0.716-1.517)			
AST (U/L)	1.073	0.714		
≥40 vs <40	(0.737-1.561)			
CA19-9 (U/mL)	1.989	0.001**		
≥40 vs <40	(1.349-2.935)			
CA125 (U/mL)	**2.002**	0.001**	**1.585**	0.030*
≥35 vs <35	**(1.350-2.967)**		**(1.045-2.404)**	
Resection Margin	1.474	0.125		
R1 vs R0	(0.898-2.417)			
Microvascular Invasion	**2.047**	<0.001**	**1.722**	0.007**
Yes vs No	**(1.403-2.987)**		**(1.157-2.562)**	
Lymph Node Metastasis	**3.264**	<0.001**	**3.194**	<0.001**
Yes vs No	**(2.226-4.788)**		**(2.127-4.796)**	
Differentiation Degree	**1.913**	0.001**	**1.704**	0.008**
Poor vs Moderate and Well	**(1.309-2.796)**		**(1.151-2.522)**	
Adjuvant therapy	**2.181**	<0.001**	**2.922**	<0.001**
No vs Yes	**(1.498-3.176)**		**(1.976-4.320)**	

BMI, Body Mass Index; ALT, Alanine Transaminase; AST, Aspartate Transaminase; CA19-9, Carbohydrate Antigen 19-9; CA125, Carbohydrate Antigen 125. *P<0.05, **P<0.01. The bold values mean that these characteristics have statistical significance. In brief, the P value of these characteristics <0.05.

### Stratification analysis of all patients

**Figure 5:**
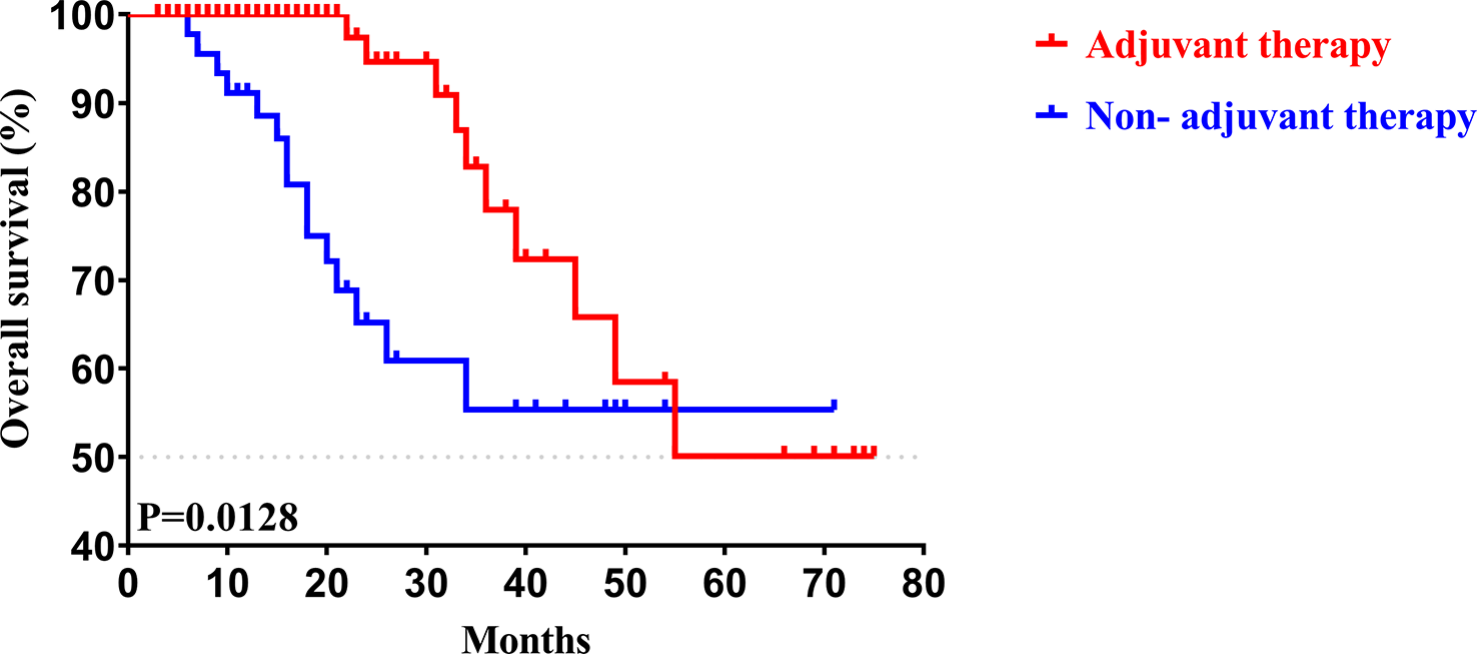
Kaplan-Meier OS curves of AJCC 8^th^ edition stage I/II patients with and without adjuvant therapy after curative resection.

**Figure 6:**
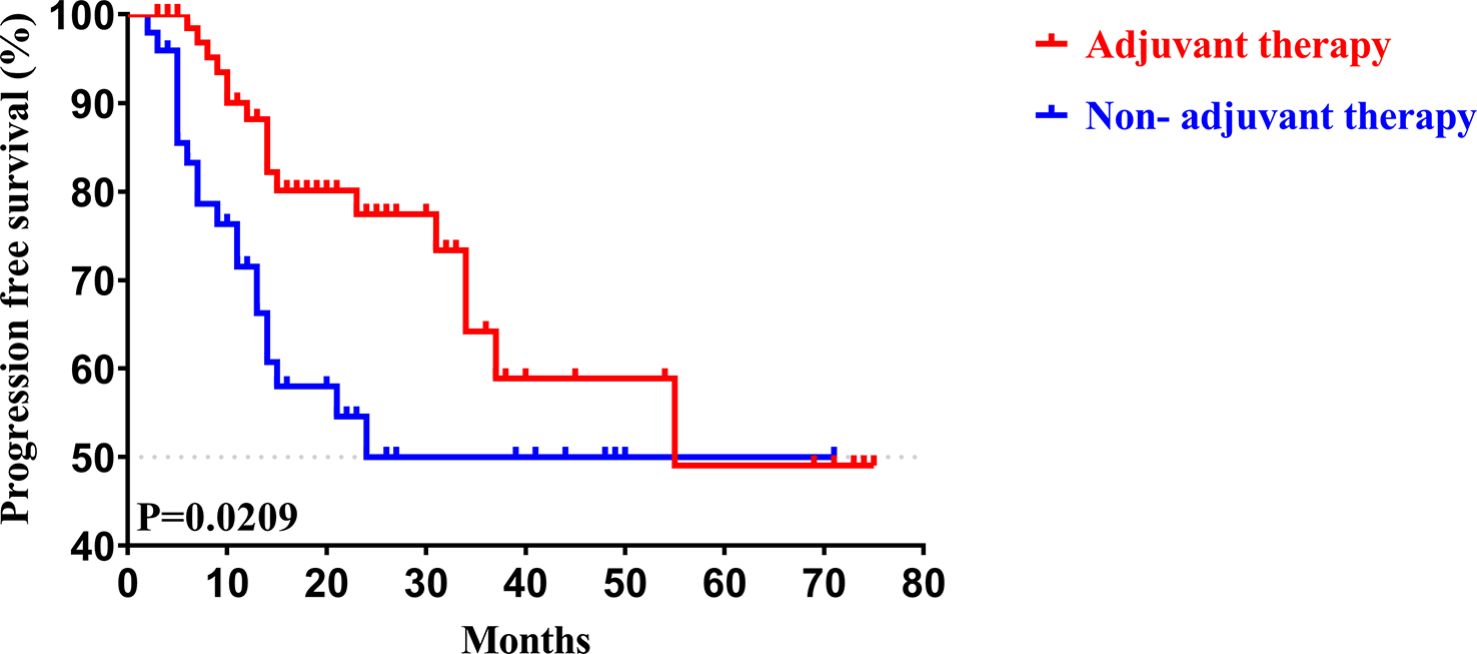
Kaplan-Meier PFS curves of AJCC 8^th^ edition stage I/II patients with and without adjuvant therapy after curative resection.

To determine whether postoperative adjuvant therapy affected the prognosis of patients with CCA in the early and advanced stages, we divided all patients into 2 groups. In the early-stage group (stages I/II), no significant differences were detected in clinicopathologic characteristics between the adjuvant therapy and non-adjuvant therapy groups (all *P* values > 0.05; **Table 5**). Survival analysis showed that the median OS (*P *= 0.0128; **Figure 5**) and the median PFS (*P *= 0.0209; **Figure 6**) differed for patients with and without adjuvant therapy. These results may indicate that postoperative adjuvant therapy could improve prognosis. Moreover, we verified that adjuvant therapy was also an independent prognostic factor for both OS (HR = 3.902; *P *= 0.003; **Table 6**) and PFS (HR = 2.502; *P *= 0.007; **Table 7**).

**Table 5:** Clinicopathologic characteristics of AJCC 8^th^ edition stage I/II patients.

Characteristics	Adjuvant therapy (n=71)	Non- adjuvant therapy (n=49)	P
Age (years)			0.195
≤60	30	15	
>60	41	34	
Sex			0.810
Male	39	28	
Female	32	21	
BMI (kg/m^2^)			0.654
<24.0	45	33	
≥24.0	26	16	
Albumin (g/dL)			0.944
<3.5	9	6	
≥3.5	62	43	
ALT (U/L)			0.757
<40	40	29	
≥40	31	20	
AST (U/L)			0.330
<40	44	26	
≥40	27	23	
CA19-9 (U/mL)			0.452
<40	44	27	
≥40	27	22	
CA125 (U/mL)			0.774
<35	58	39	
≥35	13	10	
Pathological Type			0.638
Intrahepatic	52	34	
Perihilar	16	14	
Distal	3	1	
Resection Margin			0.157
R0	68	43	
R1	3	6	
Microvascular Invasion			0.670
Yes	22	17	
No	49	32	
Differentiation Degree			0.732
Poor	28	17	
Moderate	34	27	
Well	9	5	

BMI, Body Mass Index; ALT, Alanine Transaminase; AST, Aspartate Transaminase; CA19-9, Carbohydrate Antigen 19-9; CA125, Carbohydrate Antigen 125.

**Table 6:** Univariate and multivariate analysis of overall survival in AJCC 8^th^ edition stage I/II patients.

Characteristics	Univariate analysis		Multivariate analysis	
	HR	P	HR	P
Age (years)	1.215	0.650		
>60 vs ≤60	(0.524-2.818)			
Sex	0.616	0.236		
Male vs Female	(0.276-1.374)			
BMI (kg/m^2^)	1.587	0.253		
≥24.0 vs <24.0	(0.718-3.507)			
Albumin (g/dL)	1.326	0.576		
<3.5 vs ≥3.5	(0.493-3.566)			
ALT (U/L)	2.238	0.046		
≥40 vs <40	(1.013-4.945)			
AST (U/L)	2.578	0.021*		
≥40 vs <40	(1.157-5.746)			
CA19-9 (U/mL)	1.278	0.543		
≥40 vs <40	(0.580-2.820)			
CA125 (U/mL)	0.992	0.988		
≥35 vs <35	(0.372-2.648)			
Resection Margin	**3.573**	0.012*	**3.378**	0.023*
R1 vs R0	**(1.322-9.657)**		**(1.184-9.635)**	
Microvascular Invasion	**2.289**	0.045*	**2.474**	0.035*
Yes vs No	**(1.019-5.142)**		**(1.064-5.756)**	
Differentiation Degree	**2.889**	0.014*	**3.068**	0.016*
Poor vs Moderate and Well	**(1.238-6.745)**		**(1.230-7.653)**	
Adjuvant therapy	**2.685**	0.017*	**3.902**	0.003**
No vs Yes	**(1.194-6.034)**		(1.607-9.476)	

BMI, Body Mass Index; ALT, Alanine Transaminase; AST, Aspartate Transaminase; CA19-9, Carbohydrate Antigen 19-9; CA125, Carbohydrate Antigen 125. *P<0.05, **P<0.01. The bold values mean that these characteristics have statistical significance. In brief, the P value of these characteristics <0.05.

**Table 7:** Univariate and multivariate analysis of progression free survival in AJCC 8^th^ edition stage I/II patients.

Characteristics	Univariate analysis		Multivariate analysis	
	HR	P	HR	P
Age (years)	1.029	0.935		
>60 vs ≤60	(0.523-2.023)			
Sex	1.014	0.966		
Male vs Female	(0.528-1.949)			
BMI (kg/m^2^)	1.192	0.605		
≥24.0 vs <24.0	(0.613-2.321)			
Albumin (g/dL)	1.348	0.481		
<3.5 vs ≥3.5	(0.588-3.094)			
ALT (U/L)	1.562	0.177		
≥40 vs <40	(0.817-2.987)			
AST (U/L)	1.586	0.166		
≥40 vs <40	(0.826-3.045)			
CA19-9 (U/mL)	1.818	0.070		
≥40 vs <40	(0.952-3.473)			
CA125 (U/mL)	**2.223**	0.023*	**2.233**	0.027*
≥35 vs <35	**(1.115-4.436)**		**(1.098-4.542)**	
Resection Margin	1.485	0.457		
R1 vs R0	(0.524-4.213)			
Microvascular Invasion	**3.180**	0.001**	**3.044**	0.001**
Yes vs No	**(1.627-6.217)**		**(1.548-5.985)**	
Differentiation Degree	1.907	0.058		
Poor vs Moderate and Well	(0.978-3.721)			
Adjuvant therapy	**2.102**	0.025*	**2.502**	0.007**
No vs Yes	**(1.096-4.031)**		**(1.282-4.882)**	

BMI, Body Mass Index; ALT, Alanine Transaminase; AST, Aspartate Transaminase; CA19-9, Carbohydrate Antigen 19-9; CA125, Carbohydrate Antigen 125. *P<0.05, **P<0.01. The bold values mean that these characteristics have statistical significance. In brief, the P value of these characteristics <0.05.

**Figure 7:**
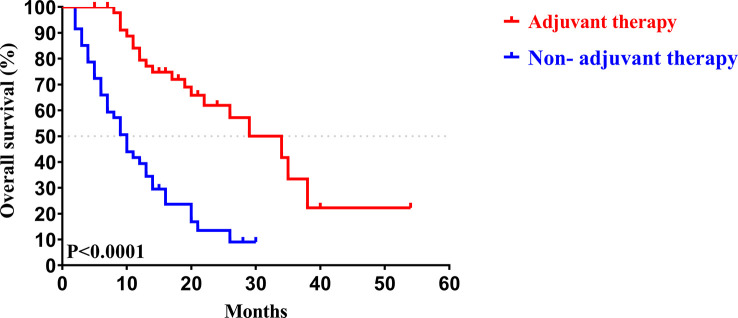
Kaplan-Meier OS curves of AJCC 8^th^ edition stage III/ IV patients with and without adjuvant therapy after curative resection.

**Figure 8:**
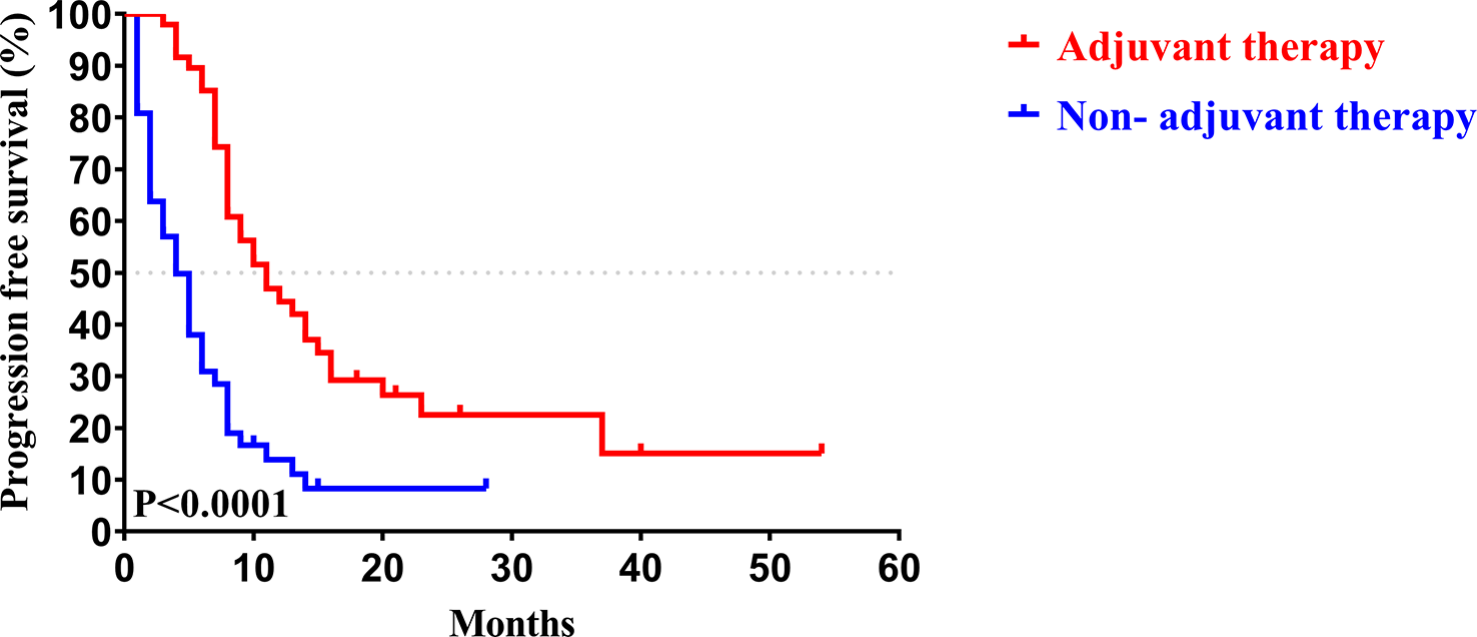
Kaplan-Meier PFS curves of AJCC 8^th^ edition stage III/ IV patients with and without adjuvant therapy after curative resection.

Similar to the early-stage group, we did not find any statistical differences in the clinicopathologic characteristics of patients in the advanced-stage (stage III/IV) between the adjuvant therapy and non-adjuvant therapy groups (**Table 8**). For the patients with and without adjuvant therapy, the median OS was 34 and 10 months (*P *< 0.001; **Figure 7**), respectively, and the median PFS was 11 and 4 months (*P *< 0.001; **Figure 8**), respectively. Furthermore, adjuvant therapy was still one of the independent prognostic factors for OS (HR = 4.551; *P *< 0.001; **Table 9**) and PFS (HR = 3.298; *P *< 0.001; **Table 10**).

**Table 8:** Clinicopathologic characteristics of AJCC 8^th^ edition stage III/IV patients.

Characteristics	Adjuvant therapy (n=48)	Non- adjuvant therapy (n=47)	P
Age (years)			0.174
≤60	24	17	
>60	24	30	
Sex			0.473
Male	26	22	
Female	22	25	
BMI (kg/m^2^)			0.942
<24.0	32	31	
≥24.0	16	16	
Albumin (g/dL)			0.145
<3.5	13	7	
≥3.5	35	40	
ALT (U/L)			0.604
<40	24	26	
≥40	24	21	
AST (U/L)			0.607
<40	23	25	
≥40	25	22	
CA19-9 (U/mL)			0.144
<40	19	12	
≥40	29	35	
CA125 (U/mL)			0.471
<35	33	29	
≥35	15	18	
Pathological Type			0.340
Intrahepatic	28	33	
Perihilar	19	14	
Distal	1	0	
Resection Margin			0.218
R0	36	40	
R1	12	7	
Microvascular Invasion			0.765
Yes	27	25	
No	21	22	
Differentiation Degree			0.657
Poor	26	23	
Moderate	20	20	
Well	2	4	

BMI, Body Mass Index; ALT, Alanine Transaminase; AST, Aspartate Transaminase; CA19-9, Carbohydrate Antigen 19-9; CA125, Carbohydrate Antigen 125.

**Table 9:** Univariate and multivariate analysis of overall survival in AJCC 8^th^ edition stage III/IV patients.

Characteristics	Univariate analysis		Multivariate analysis	
	HR	P	HR	P
Age (years)	1.095	0.524		
Male vs Female	(0.650-1.842)			
BMI (kg/m^2^)	1.032	0.908		
≥24.0 vs <24.0	(0.603-1.767)			
Albumin (g/dL)	1.129	0.704		
<3.5 vs ≥3.5	(0.604-2.108)			
ALT (U/L)	0.955	0.862		
≥40 vs <40	(0.568-1.605)			
AST (U/L)	0.940	0.816		
≥40 vs <40	(0.560-1.578)			
CA19-9 (U/mL)	1.755	0.062		
≥40 vs <40	(0.973-3.166)			
CA125 (U/mL)	**1.684**	**0.054**	**1.810**	0.030*
≥35 vs <35	**(0.991-2.862)**		**(1.060-3.090)**	
Resection Margin	0.850	0.622		
R1 vs R0	(0.446-1.619)			
Microvascular Invasion	1.243	0.415		
Yes vs No	(0.737-2.095)			
Differentiation Degree	1.346	0.262		
Poor vs Moderate and Well	(0.801-2.263)			
Adjuvant therapy	**4.403**	<0.001**	**4.551**	<0.001**
No vs Yes	**(2.452-7.909)**		**(2.525-8.201)**	

BMI, Body Mass Index; ALT, Alanine Transaminase; AST, Aspartate Transaminase; CA19-9, Carbohydrate Antigen 19-9; CA125, Carbohydrate Antigen 125. *P<0.05, **P<0.01. The bold values mean that these characteristics have statistical significance. In brief, the P value of these characteristics <0.05.

**Table 10:** Univariate and multivariate analysis of progression free survival in AJCC 8^th^ edition stage III/IV patients.

Characteristics	Univariate analysis		Multivariate analysis	
	HR	P	HR	P
Age (years)	0.932	0.762		
>60 vs ≤60	(0.590-1.473)			
Sex	1.091	0.711		
Male vs Female	(0.689-1.726)			
BMI (kg/m^2^)	1.492	0.094		
≥24.0 vs <24.0	(0.934-2.383)			
Albumin (g/dL)	0.651	0.162		
<3.5 vs ≥3.5	(0.357-1.188)			
ALT (U/L)	0.682	0.107		
≥40 vs <40	(0.428-1.086)			
AST (U/L)	0.661	0.079		
≥40 vs <40	(0.416-1.049)			
CA19-9 (U/mL)	1.252	0.375		
≥40 vs <40	(0.763-2.054)			
CA125 (U/mL)	1.426	0.147		
≥35 vs <35	(0.882-2.306)			
Resection Margin	0.876	0.653		
R1 vs R0	(0.493-1.558)			
Microvascular Invasion	1.103	0.676		
Yes vs No	(0.696-1.748)			
Differentiation Degree	**1.593**	**0.051**	**1.950**	0.007**
Poor vs Moderate and Well	**(0.997-2.545)**		**(1.203-3.161)**	
Adjuvant therapy	**2.906**	<0.001**	**3.298**	<0.001**
No vs Yes	**(1.803-4.684)**		**(2.019-5.387)**	

BMI, Body Mass Index; ALT, Alanine Transaminase; AST, Aspartate Transaminase; CA19-9, Carbohydrate Antigen 19-9; CA125, Carbohydrate Antigen 125. **P<0.01. The bold values mean that these characteristics have statistical significance. In brief, the P value of these characteristics <0.05.

Thus, we concluded that postoperative adjuvant therapy could effectively improve the prognosis for patients with CCA in both the early and advanced stages.

## Discussion

CCA is the second most common primary hepatic carcinoma after hepatocellular carcinoma (HCC) and has low survival and high recurrence rates (3,4). The 2019 American Society of Clinical Oncology (ASCO) clinical practice guideline recommends treating patients with adjuvant capecitabine for a duration of 6 months following curative resection of CCA based on the partial benefit reported in the BILCAP trial (16). However, the intention-to-treat analysis conducted in the BILCAP trial failed to show the benefit of OS (the primary endpoint; HR 0.81; *P* = 0.097) (14). This finding was inconsistent with other studies and led to confusion in treating patients with CCA after surgery (17). In our study, adjuvant therapy was associated with improved survival in patients with CCA, which prolonged the median OS and PFS compared with surgery alone. Furthermore, univariate and multivariate analysis of OS and PFS revealed that adjuvant therapy was one of the independent prognostic factors and was closely related to survival for all patients with CCA at the early or advanced stages. More importantly, with the help of stratified analysis, we verified that patients at advanced stages could benefit from adjuvant therapy, and we found a beneficial effect in patients at the early stages of CCA. Overall, these data adequately suggested that, with routine use of adjuvant therapy, patients could achieve great prognoses in the early or advanced stages of CCA.

The role of adjuvant therapy for CCA is still not clearly defined due to equivocal evidence in the relevant literature. Several meta-analyses have examined the benefit of adjuvant therapy, but the results are conflicting (18–22). The meta-analysis reported by Horgan et al. (18), which analyzed 20 studies involving 6712 patients, showed no significant improvement in OS with any type of adjuvant therapy compared with surgery alone (odds ratio 0.74; *P *= 0.06). However, another meta-analysis conducted by Rangarajan et al. (20), which examined 35 studies, drew the opposite conclusion (*P *< 0.001; HR 0.74). Although the current evidence is largely limited, overall, most retrospective studies published in the past few years have demonstrated that adjuvant therapy following surgery can help patients with CCA achieve greater prognosis (23–27). Perhaps most significantly, the BILCAP study published its most recent follow-up data in 2022 (28): there was no difference in median OS in the capecitabine group compared with the control group (HR 0.84; 95% CI: 0.67–1.06) in the intention-to-treat analysis; however, in a protocol-specified sensitivity analysis, a significant difference was observed (HR 0.74; 95% CI: 0.59–0.94). Taken together, these findings indicate that treatment with adjuvant therapy following surgery for patients with CCA should be fully considered, and capecitabine may be the first choice.

Prognostic factors may help identify high-risk patients and select appropriate patients for adjuvant therapy, so we further analyzed OS- and PFS-related independent risk factors for patients with CCA. We found microvascular invasion, lymph node metastasis, and differentiation degree to be the independent risk factors of OS and PFS. These results are consistent with other studies (29–33). For instance, Rao et al. (29) showed that specific risk factors, including vascular invasion and differentiation degree, were associated with the OS and PFS of CCA after curative resection. Zhang et al. (31) reported that lymph node metastasis was associated with shorter long-term survival (*P *< 0.001) and recurrence-free survival (RFS; *P*< 0.001) for patients with iCCA. Additionally, a study conducted by Huang et al. (33) demonstrated that vascular invasion, differentiation degree and lymph node involvement were independent predictors for OS and RFS in iCCA after curative resection.

Some tumor markers, such as CA 19-9, may be used to determine postoperative prognosis and as a diagnostic indicator (34–37). Asaoka et al. (35) found that a preoperative CA19-9 value of no more than 37 U/ml was associated with better OS and RFS. Data from a study by Tella et al. (36) also showed preoperative CA19-9 (> 38 U/ml) to be associated with poor prognosis and was regarded as an independent prognostic factor affecting OS. Besides CA19-19, serum CA125, firstly discovered and identified as tumor marker of ovarian cancer in 1981 by Niloff et al. (38), is not only used in the diagnosis of varieties of tumors, but an effective prognostic biomarker of tumors (39). Xu et al. (40) found that preoperative serum CA125 served as a good tumor marker to predict prognosis of pCCA after curative resection. Thus, combined the data from our study, preoperative CA19-9 was considered an independent prognostic factor of OS and preoperative CA 125 was an independent risk factor of PFS.

To the best of our knowledge, this study is the first to demonstrate the benefit of adjuvant therapy for patients with CCA after surgery both in the early and advanced stages. We identified a series of clinicopathologic characteristics to predict prognosis and select high-risk patients who might benefit from clinical treatment. However, several limitations must be acknowledged in this study. First, the single-center retrospective design of this study lends itself to potential selection bias. A greater number of patients with CCA after resection from other medical centers should be included in future studies to validate our results. Second, the type of adjuvant therapy following surgery for CCA varied in this study, and we did not analyze the correlation between the adjuvant therapy regimen and the prognosis of CCA due to the paucity of data from other treatment regimens. Third, the cutoff value of CA 19-9 and CA125 used different definitions compared to previous studies. Further high-quality studies should be conducted to address these limitations.

## Conclusion

Our study retrospectively analyzed 215 patients with CCA after radical resection and confirmed that adjuvant therapy following resection, as an independent prognosis factor, significantly improved the prognosis (OS and PFS) of patients with early- or advanced-stage CCA. Microvascular invasion, lymph node metastasis, and differentiation degree were independent prognostic factors for both OS and PFS. Preoperative CA19-9 were independent risk factors affecting OS. Preoperative CA125 was an independent risk factor associated with PFS. These factors may be valuable in screening patients most suited to adjuvant therapy.

## Data availability statement

The original contributions presented in the study are included in the article/supplementary material. Further inquiries can be directed to the corresponding authors.

## Ethics statement

The study was in accordance with the ethical guidelines of the 1975 Declaration of Helsinki. Ethical approval was obtained from the Ethics Committee of the Second Affiliated Hospital, School of Medicine, Zhejiang University.

## Author contributions

Conception and design: WW and YD. Collection and assembly of data: XH, JT, JX, BY, TL, YZ and KC. Data analysis: ZS, XH and WY. Manuscript writing: ZS and XH. Final approval of manuscript: WW and YD. All authors contributed to the article and approved the submitted version.
